# Molecule-Resolved Visualization of Particulate Matter on Human Skin Using Multimodal Nonlinear Optical Imaging

**DOI:** 10.3390/ijms22105199

**Published:** 2021-05-14

**Authors:** Eun-Soo Lee, Suho Kim, Sang-Won Lee, Jinsang Jung, Sung Hoon Lee, Hye-Won Na, Hyoung-June Kim, Yong Deog Hong, Won Seok Park, Tae Geol Lee, Dong-Gyu Jo, Se-Hwa Kim

**Affiliations:** 1 AMOREPACIFIC Research and Development Center, Yongin 17074, Korea; soopian82@gmail.com (E.-S.L.); imstrong20@amorepacific.com (S.H.L.); serina@amorepacific.com (H.-W.N.); leojune@amorepacific.com (H.-J.K.); hydhong@amorepacific.com (Y.D.H.); wspark@amorepacific.com (W.S.P.); 2 School of Pharmacy, Sungkyunkwan University, Suwon 16419, Korea; shkim27@kriss.re.kr (S.K.); jodg@skku.edu (D.-G.J.); 3 Safety Measurement Institute, Korea Research Institute of Standards and Science, Daejeon 34113, Korea; swlee76@kriss.re.kr (S.-W.L.); tglee@kriss.re.kr (T.G.L.); 4 Department of Medical Physics, University of Science and Technology, Daejeon 34113, Korea; 5 Gas Metrology Group, Division of Chemical and Biological Metrology, Korea Research Institute of Standards and Science, Daejeon 34113, Korea; jsjung@kriss.re.kr

**Keywords:** particulate matter, nonlinear optical imaging, spatial analysis, human skin

## Abstract

Precise measurement of particulate matter (PM) on skin is important for managing and preventing PM-related skin diseases. This study aims to directly visualize the deposition and penetration of PM into human skin using a multimodal nonlinear optical (MNLO) imaging system. We successfully obtained PM particle signals by merging two different sources, C–C vibrational frequency and autofluorescence, while simultaneously visualizing the anatomical features of the skin via keratin, collagen, and elastin. As a result, we found morphologically dependent PM deposition, as well as increased deposition following disruption of the skin barrier via tape-stripping. Furthermore, PM penetrated more and deeper into the skin with an increase in the number of tape-strippings, causing a significant increase in the secretion of pro-inflammatory cytokines. Our results suggest that MNLO imaging could be a useful technique for visualizing and quantifying the spatial distribution of PM in ex vivo human skin tissues.

## 1. Introduction

High levels of ambient particulate matter (PM) in pollutants due to industrialization and urbanization have been associated with outbreaks of human diseases. A complex mixture of solid and liquid particles, PM contains nitrates, sulfates, hydrocarbons, benzene, toluene, metals, and other substances, and is classified by size as coarse (mean diameter <10 μm), fine (PM_2.5_, mean diameter <2.5 μm), and ultrafine (mean diameter <0.1 μm). PM has been reported to cause various health problems, including respiratory disease, cardiovascular diseases such as hypertension, and neurotoxicity [1,2,3,4]. Moreover, long-term exposure to PM has been shown to effect a decrease in life expectancy [5].

Particulate matter also exerts detrimental effects on human skin by inducing the acceleration of inflammatory skin diseases such as atopic dermatitis, acne, and psoriasis [6,7,8]. Additionally, chronic PM exposure is associated with extrinsic skin aging and an increase in wrinkles and pigment spots [9]. However, it should be noted that these previous reports are epidemiologic studies. Cell-based experiments, on the other hand, have revealed that PM_2.5_ significantly alters the expression of genes related to epidermal development, differentiation, and inflammation in normal human epidermal keratinocytes [10]. In addition, benzo(a)pyrene (BaP), a type of polycyclic aromatic hydrocarbon (PAH) in PM, enhances oxidative stress-mediated inflammatory cytokines in human keratinocytes [11]. Due to the limitations of in vitro systems, though, PM effects need to be understood in a spatial environment.

In terms of skin penetration, while a number of studies have observed PM in various epidermal layers, details about the process of PM penetration into the stratum corneum (SC), which is the first interface between the human body and its environment, have yet to be explored. Generally, percutaneous penetration of PM-like microparticles is related to various factors such as age, the integrity of the skin barrier, and the anatomic site [12]. As for the penetration pathway, two previous studies have reported that microparticles can penetrate skin through the hair transfollicular route [13,14]. Techniques to study PM penetration traditionally include transmission electron microscopy (TEM), which has been used to show penetration into the barrier-disrupted epidermis of mice [15], as well as energy dispersive X-ray spectroscopy (EDS) and Raman spectroscopy, which have been applied to visualize the spatial distributions of PM [16,17,18]. However, such approaches are insufficient to directly visualize and precisely quantify the accumulation of PM.

Nonlinear optics (NLO) is a useful technique that in principle enables the imaging of a non-labeled target biomolecule in terms of its molecular structures via atomic vibrations [19]. We have previously developed the multimodal nonlinear optical (MNLO) imaging system, which combines coherent anti-Stokes Raman scattering (CARS), second harmonic generation (SHG), and two-photon excitation fluorescence (TPEF), and have applied it to various label-free ex vivo investigations [20,21,22]. The primary advantage of the MNLO system is that it provides for the simultaneous visualization of the target molecule as well as the features of the tissue sample; CARS is in charge of molecular structure-specific visualization, while SHG and TPEF provide extracellular matrix information.

In the present study, we postulate that PM penetrates deeper into damaged skin, consequently accelerating human skin diseases. To verify the penetration of PM, we employ our developed MNLO system based on the inherent molecular structures of PM and the skin. This study is performed on human skin biopsy samples using collected airborne PM_2.5._ We obtain precise spatial information of the PM from *en face* three-dimensional (3D) MNLO images, with which we can consider the effect of skin damage on the level of PM penetration. We additionally measure inflammatory responses and find that they are increased in damaged skin containing infiltrated PM. Thus, with direct evidence visualizing both deposited and penetrated PM, the results of this work support the hypothesis that damaged skin is more sensitive to PM exposure, which could eventually lead to worsened skin diseases or other conditions.

## 2. Results

### 2.1. Demonstration of MNLO Imaging of Airborne PM_2.5_

**Figure 1:**
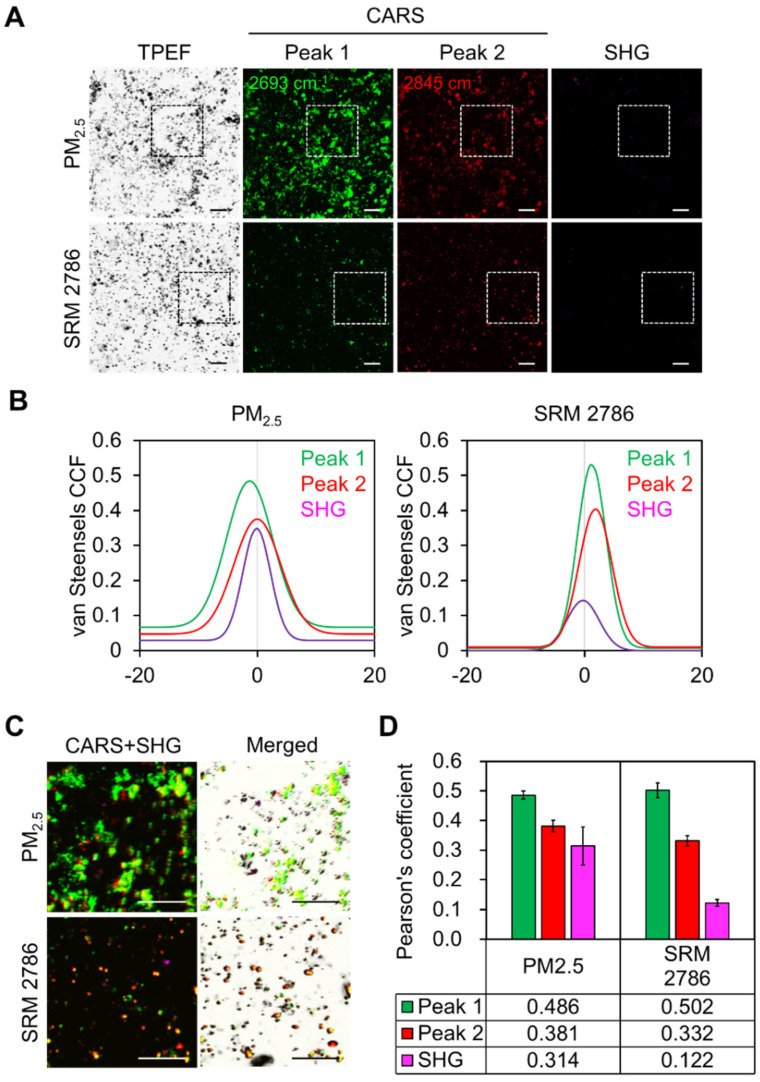
Characterization of PM_2.5_ and optimization of MNLO imaging. (**A**) MNLO images of airborne-collected PM_2.5_ and SRM 2786. Images from TPEF (dark gray), SHG (magenta), and CARS (green and red) were obtained from the same region. Two wavelengths (peak 1 and peak 2) of CARS were assigned as the C–C and C–H vibrational modes at ~2693 ± 20 cm^−1^ and at ~2840 ± 20 cm^−1^, respectively. The square indicated by the dotted line indicates the enlarged images of ROI in (**C**). Scale bars, 20 μm. (**B**) Overlap of the two CARS signals or the SHG signal by van Steensel’s CCF based on the TPEF signal f0rom PM_2.5_ and SRM 2786. (**C**) Enlarged images of the CARS (peak 1 and peak 2), the SHG, and the TPEF of the ROIs shown in (**A**). Scale bars, 20 μm. (**D**) Pearson’s coefficient analysis of the CARS (peak 1 and peak 2) and the SHG based on the TPEF signal from PM_2.5_ and SRM 2786.

To demonstrate the MNLO imaging of PM_2.5,_ we optimized the wavelengths of the lasers in CARS based on its molecular vibrational characteristics. Since PM_2.5_ is a complex mixture of carbonaceous materials [23], two wavelengths (peak 1 and peak 2) were assigned for the C–C and C–H vibrational modes at ~2693 ± 20 cm^−1^ and at ~2840 ± 20 cm^−1^, respectively. We interpret that CARS peak 1 corresponds to the two-dimensional (2D) band of Raman spectra in carbon nanotubes [24,25] and that CARS peak 2 indicates the C–H vibrational mode that is expected to produce a strong signal when carbon complexes such as PAHs are hydrated. The airborne PM_2.5_ was validated with the standard reference material (SRM) 2786 from NIST via MNLO imaging. As shown in Figure 1A, strong intensities were measured at peaks 1 and 2, indicating C–C and C–H molecular bonding enrichment in airborne PM_2.5_ and SRM 2786. Along with CARS in MNLO, TPEF also showed a high intensity of both PM sources resulting from the fluorescence of PAHs. To confirm the reliability of the multimode signals from the PM sources, we analyzed the pixel colocalization of all images using van Steensel’s cross-correlation of PM [26]. As shown in Figure 1B, two peaks of CARS and SHG are highly overlapped with TPEF, indicating an identical source. Furthermore, MNLO imaging is capable of sensitively detecting the multiple components of the diverse mixture conditions of PM, as shown in Figure 1C,D. Therefore, we conducted all MNLO imaging in this work using peaks 1 and 2 of CARS and SHG. In the subsequent experiments, the airborne collected PM_2.5_ (hereafter, PM_2.5_) was used in our investigations with human skin biopsy samples.

### 2.2. Label-Free Visualization of PM_2.5_ on Human Skin by En Face MNLO 3D Imaging: The Necessity of CARS for Discriminating PM from Skin Autofluorescence

**Figure 2:**
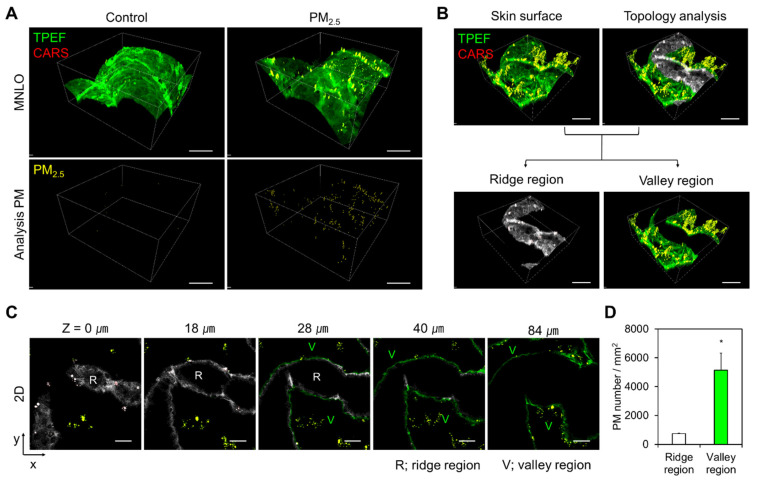
*En face* MNLO 3D imaging of PM_2.5_ in human skin biopsy samples. (**A**) *En face* MNLO images of the label-free stratum corneum (TPEF, green) and PM_2.5_ (CARS, red in the upper panels and yellow in the lower panels) in human skin biopsy samples after PM_2.5_ exposure. Consecutive *en face* MNLO image slices were reconstructed in 3D. The measurement volume was 420 (*x*) × 420 (*y*) × 200 (*z*) μm^3^. Scale bars, 100 μm. (**B**) Topological analysis of the *en face* MNLO 3D images. Ridge and valley regions of the skin microrelief are indicated in gray and green, respectively. Scale bars, 100 μm. (**C**) *En face* MNLO image slices from indicated *z*-depths after topological analysis. Scale bars, 60 μm. (**D**) Number of PM_2.5_ particles in the ridge and valley regions of the skin microrelief (mean ± S.E.M., * *p* < 0.05; two-tailed Student’s *t*-test).

To fully apply the advantages of MNLO imaging, we first assigned the CARS modality for the molecular features of the PM_2.5_, as explained above, and then utilized the TPEF and SHG modalities for elastin and collagen, respectively, for the simultaneous analysis of skin anatomy. To do this, human skin biopsy samples were exposed to PM_2.5_ for 24 h, and then *en face* 3D imaging was conducted without pretreatment processes. Figure 2A and Supplementary Video S1 show representative *en face* MNLO 3D imaging results with a volume of 420 (*x*) × 420 (*y*) × 200 (*z*) μm^3^. The hexagonal structure of the SC was imaged using the autofluorescence of keratin by TPEF. To identify the source of the fluorescence signal from PM_2.5_, we investigated whether PAHs, as a main component of PM_2.5_ [27], generate fluorescence signals. As shown in Supplementary Figure S1, PAHs were imaged in small particle form, displaying both autofluorescence with a wavelength of 495–540 nm by TPEF and a C–C vibrational signal at 2693 cm^−1^ by CARS. Given this, we assumed that a substantial amount of the TPEF imaging signals from PM_2.5_ derive from PAHs. To distinguish the autofluorescence signals from the skin and from the PM_2.5_, it is necessary to detect the specific molecular vibrations of the PAHs via CARS. As shown in the lower panels of Figure 2A, PM_2.5_ on the skin surface needs to be visualized by both CARS and TPEF.

### 2.3. Wide-View 3D MNLO Imaging of PM_2.5_ on Skin Showing Morphological Dependency

To demonstrate topological analysis, we performed wide-view 3D MNLO imaging of human skin samples and found a topological tendency for PM_2.5_ to deposit more in the valleys of the skin microrelief than on the ridges [28]. As shown in Figure 2B, we divided the 3D-rendered MNLO images of the skin surface into ridge (gray) and valley (green) regions based on the morphology of the skin microrelief. The two regions were divided at a z-depth of approximately 30 μm, as shown in Figure 2C. According to the quantification results for each region in Figure 2D, PM_2.5_ was predominantly deposited in the valleys compared with the ridges. This result indicates that MNLO imaging-based PM_2.5_ analysis could be useful to more precisely measure PM_2.5_ in a *z*-depth manner.

### 2.4. Volumetric Quantification of PM_2.5_ Deposition between Healthy and Damaged Skin

**Figure 3:**
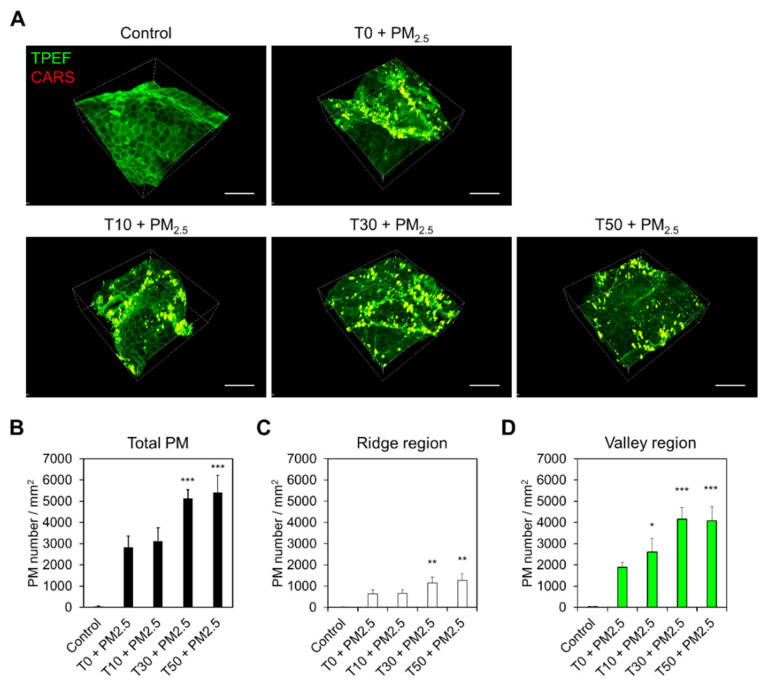
Visualization of PM_2.5_ deposition on the skin surface by skin barrier disruption. (**A**) *En face* MNLO 3D images of the label-free stratum corneum (TPEF, green) and PM_2.5_ (CARS, red) in PM_2.5_-exposed human skin biopsy samples after tape-stripping (T). T10, T30, and T50 indicate strippings of 10, 30, and 50 times, respectively. Scale bars, 100 μm. (**B**–**D**) Number of PM_2.5_ particles on the skin surface, ridge, and valley regions by the number of tape-strippings (mean ± S.E.M., * *p* < 0.05, ** *p* < 0.01, *** *p* < 0.001; two-tailed Student’s *t*-test).

Employing 3D MNLO imaging, we quantitatively analyzed the deposition of PM_2.5_ on damaged compared to healthy skin. The tape-stripping (T) method was applied to human skin biopsy samples in order to disrupt the skin barrier function of the SC [29]. The level of damage was controlled by the number of strippings; in this work, 0, 10, 30, and 50 strippings were applied (T0, T10, T30, and T50, respectively) prior to exposure to PM_2.5_. Representative images in Figure 3A show the results of visualizing human skin with deposited PM_2.5_ using *en face* MNLO 3D imaging. In the quantitative analysis in Figure 3B–D, the deposition of PM_2.5_ in the T30 sample was significantly increased (by approximately two-fold) compared to T0 and T10. Interestingly, as the number of strippings increased, not only did the total amount of PM_2.5_ deposition increase, but the proportion of deposition in the valley regions increased as well.

### 2.5. Percutaneous Penetration of PM_2.5_ Deepens with Severe Skin Damage

**Figure 4:**
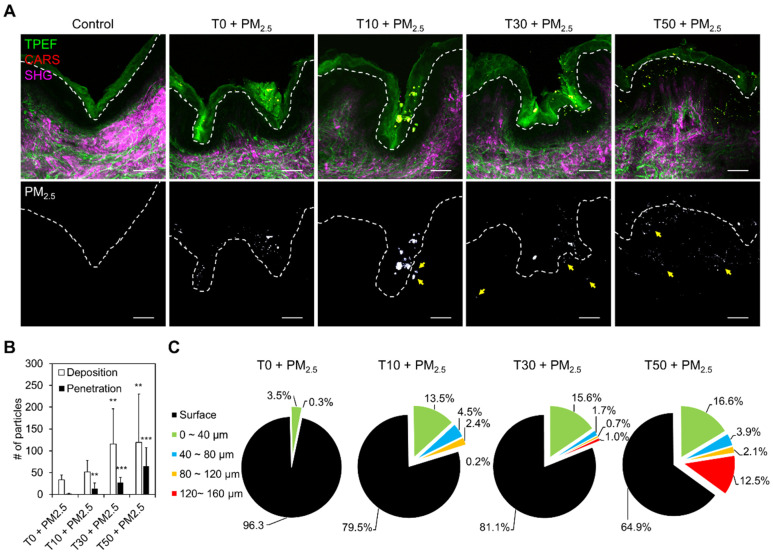
Analysis of PM_2.5_ penetration by skin barrier disruption. (**A**) MNLO images of label-free elastin and keratin (TPEF, green), PM_2.5_ (CARS, red), and collagen (SHG, magenta) in cross-sectioned PM_2.5_-exposed human skin biopsy samples after tape-stripping (T). The dashed lines mark the boundary between the stratum corneum and the stratum spinosum. Scale bars, 60 μm. (**B**) Number of PM_2.5_ particles deposited or penetrated in the cross-sectioned samples (mean ± S.E.M., ** *p* < 0.01, *** *p* < 0.001; two-tailed Student’s *t*-test). (**C**) PM_2.5_ penetration depths by number of strippings, from measuring the distance between the penetrated PM_2.5_ and the dashed lines.

To investigate the percutaneous penetration of PM_2.5_, we performed cross-sectional MNLO imaging on human skin samples considering the same levels of skin damage as in the previous section. As shown in Figure 4A and Supplementary Video S2, TPEF (green) and SHG (magenta) signals highlight the anatomy of the skin without any staining method; TPEF signals indicate keratin in the SC and elastin in the connective tissue, while SHG indicates collagen in the connective tissue. To clearly visualize penetrated PM_2.5_, CARS intensity is shown in the black/white scale with dotted lines that divide the SC and the stratum spinosum (SS) in the lower panels of Figure 4A. In the quantitative analysis, the region above the dotted line, or SC, was regarded as PM_2.5_ deposition, and the region below, or SS, was counted as penetration. Like the previous *en face* analysis, deposition significantly increased as the number of strippings increased from T30 to T50 (white bar in Figure 4B). However, even at T10, the penetration of PM_2.5_ could already be visualized (black bar in Figure 4B). This percutaneous penetration was further investigated in terms of the depth from the top of the SS measured in 40 μm sections (Figure 4C). At a shallow depth (<40 μm *z*-depth), PM_2.5_ penetration was maintained regardless of the level of skin damage. Remarkably, in the most severely damaged sample, T50, a substantial percentage of PM_2.5_ penetrated to a depth of 120–160 μm, as shown in red in Figure 4C.

### 2.6. Increased Inflammatory Gene Expression Induced by PM_2.5_ Exposure with Skin Barrier Disruption

**Figure 5:**
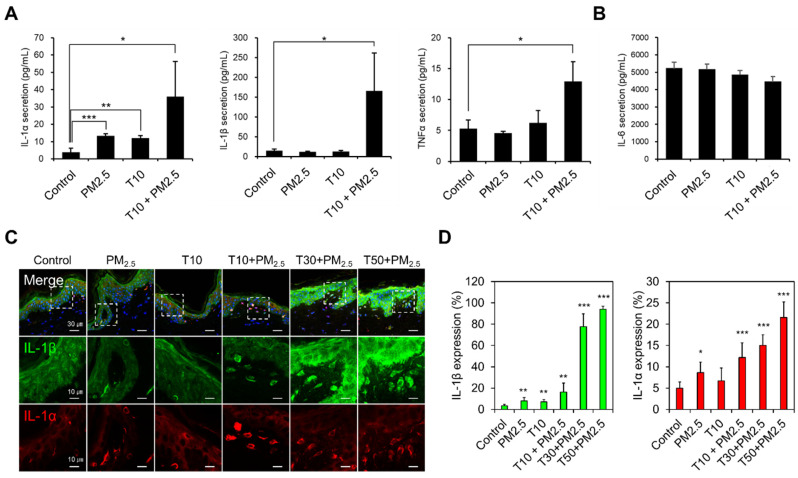
Inflammatory response induced by PM_2.5_ exposure and skin barrier disruption. (**A**,**B**) Secreted cytokines (IL-1α, IL-1β, TNF-α, and IL-6) measured in culture medium by ELISA. (**C**) Confocal images of cross-sectioned PM_2.5_-exposed human skin biopsy samples after tape-stripping (T). IL-1β (green) and IL-1α (red) were stained with antibodies, and nuclei were counter-stained with DAPI (blue). The white dashed boxes in the merged images (upper row) are enlarged in the separated images (lower rows). Scale bars, 30 μm for the merged images and 10 μm for the enlarged images. (**D**) The cells with a high expression of IL-1β and IL-1α were quantified in each condition (mean ± S.E.M., * *p* < 0.05, ** *p* < 0.01, *** *p* < 0.001; two-tailed Student’s *t*-test).

PM has been reported to cause oxidative stress and inflammatory response in skin [30]. To further examine the biological responses of penetrated PM_2.5_, we performed ELISA and immunofluorescence staining of ex vivo human skin tissue considering damage (T10) and PM_2.5_ exposure. First, several cytokines secreted to the culture medium were measured by ELISA. In both PM_2.5_-treated and non-damaged skin (control), IL-1α was increased, while IL-1β and TNF-α were unchanged. However, in T10-damaged skin, IL-1α, IL-1β, and TNF-α were significantly increased (Figure 5A). IL-6 was not changed by either PM_2.5_ exposure or T10 (Figure 5B). To confirm the inflammatory responses induced by penetrated PM_2.5_, expression of the cytokines inside the tissue was confirmed by immunofluorescence staining. Results show that IL-1α- and IL-1β-positive cells were found underneath the SC in PM_2.5_-treated T10, T30, and T50 (Figure 5C). In PM_2.5_-treated T30 and T50, IL-1β-positive cells increased significantly compared to PM_2.5_-treated T10, but IL-1α-positive cells did not (Figure 5D). This prominent change in IL-1β is expected as a result of overlapping effects of skin damage and PM exposure, suggesting that IL-1β is a cytokine that leads to an inflammatory response. Such results indicate that PM_2.5_ exposure may lead to increased inflammatory responses in damaged compared to healthy skin.

## 3. Discussion

Even though the skin represents the largest organ with substantial direct contact to the atmosphere, measuring PM on the skin as a potential harmful factor has, to date, not been straightforward, and thus the toxicity of PM to the skin is not yet fully understood. Here, the principle of multimodal nonlinear optics was utilized for the direct 3D visualization of both adhesive and penetrated PM_2.5_ on human skin without labeling. Depending on the skin morphology, PM_2.5_ was differentially accumulated in the valleys and ridges of the skin microrelief. The application of MNLO was also able to demonstrate that damaged skin could be more susceptible to PM_2.5_ by deeper penetration toward the dermis compared to healthy skin.

Nonlinear optics such as TPEF and SHG has already been applied in dermatological studies, enabling deep optical imaging [31]. Both of these optical techniques are useful to provide skin morphological information, since autofluorescent biomolecules (keratin, melanin, elastin, etc.) can be imaged by TPEF and anisotropic fibrous molecules such as collagen can be visualized by SHG. Despite these advantages, nonlinear optics technologies have yet to be more widely expanded in dermatological research. In the case of TPEF, as endogenous fluorescent molecules can be imaged with exogenous target molecules, signal interferences need to be technically figured out. Likewise in our study, PM fluorescence needs to be selectively distinguished from autofluorescent biomolecules [32]. We therefore additionally installed CARS to detect the intrinsic molecular fingerprints of PM giving the Raman peaks, so that PM can be selectively imaged along with the morphological features of the skin without interference (Supplementary Figure S1) [24,25]. Unlike conventional Raman scattering mapping that takes several hours, CARS provides fast scanning in under 1 s, possibly opening the door to other applications.

To date, it has been taken for granted that PM infiltrates the skin through transfollicular routes, with no direct evidence for the morphologically dependent deposition and penetration of PM yet observed. Although PM penetration into skin was demonstrated by Jin et al. through TEM [15], such sectional views are limited to supporting a good conformational analysis of the skin. In our *en face* 3D MNLO imaging, we showed that PM_2.5_ significantly accumulated in the valley regions compared to the ridge regions of the skin microrelief (Figure 2). This morphological dependency might provide supporting evidence for PM_2.5_ infiltration through transfollicular routes.

**Figure 6:**
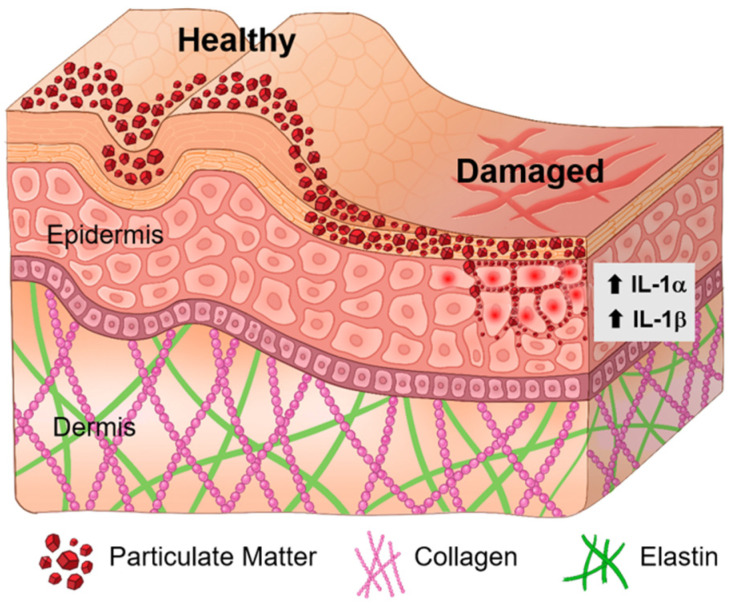
Model of PM deposition and penetration in response to skin barrier damage. This diagram shows 3D spatial deposition and penetration of PM depending on skin barrier disruption. In healthy condition, the topology-dependent distribution of PM is identified. In damaged skin, a substantial amount of PM infiltrated in terms of skin barrier damage level. Inflammatory IL-1α- and IL-1β-positive cells were found underneath the SC in PM_2.5_-treated damaged skin.

Skin conditions in which the skin barrier is compromised, such as atopic dermatitis, could be worsened by exogenous factors [33]. To investigate PM effects, we employed human skin biopsy samples ranging from healthy to severely damaged conditions by the tape-stripping technique. Since over 30 strippings remove about 30% of the epidermis [29], meaning the almost complete removal of the SC, we tested 10, 30, and 50 strippings for comparison with no stripping. The results showed the following distinctive characteristics. First, the total deposition of PM_2.5_ increased with the number of strippings from the loss of the SC (Figure 3). Although PM was treated under the same conditions, the accumulated amount of PM on the skin particularly increased in T30 and T50, implying that the SC might provide defensive physiological functions. Second, the amount of penetrated PM_2.5_ also increased with the number of strippings (Figure 4); however, a substantial amount (about 13–16%) was consistently detected at <40 μm depth in all tape-stripping samples. This depth could be an important inner border possibly generated by the increased cohesion between cells in deeper layers [34]. Remarkably, in the T50 sample, about 12.5% of the PM_2.5_ penetrated to a depth of 120–160 μm; since T50 represents severely damaged skin, this result implies that serious skin conditions may have an increased sensitivity to PM exposure. Third, the sizes of the individual infiltrated particles were relatively smaller than those by deposition. It is worth noting that the infiltrated PM_2.5_ accumulated along the line of the dermis layer. Although the penetration mechanism needs to be studied further, smaller-sized PM_2.5_ particles might more easily pass through the cohesive SS. To our knowledge, this is the first demonstration of the 3D spatial distribution of PM_2.5_ both deposited on and penetrated into healthy and damaged human skin. Overall results are summarized in a schematic diagram in Figure 6. 

## 4. Materials and Methods

### 4.1. PM_2.5_ Preparation and Analysis

PM_2.5_ was collected from the atmosphere at the rooftop of the Amorepacific Corporation R&D building located in Yongin, Republic of Korea (37°15′ N 127°06′ E) during January–April 2018. A high-volume air sampler (TE6070, Tisch Environmental, Cleves, OH, USA) was operated at a flow rate of 1.13 m^3^/min for 24 h and a polytetrafluoroethylene (PTFE) filter (Zefluor, Pall Corporation, Ann Arbor, MI, USA) was placed in front of the instrument to collect PM_2.5_. After extraction–sonication–evaporation, the extracted PM_2.5_ was resuspended in 20% EtOH and stored at –20 °C until use. SRM 2786 was purchased from Sigma Aldrich (St. Louis, MO, USA).

### 4.2. Human Skin Tissue and Exposure to PM_2.5_

Human skin explant NativeSkin^TM^ models purchased from Genoskin (Toulouse, France) were collected from punch biopsies of non-sun-exposed skin of healthy donors. After plastic surgery, tape-stripping was performed, and then the tissues were embedded in a matrix and fixed in a cell culture insert. Informed consent was obtained from the donors and the experimental use of the skin biopsies was approved by the Comité de Protection des Personnes (CPP) in France and the ECU Human Research Ethics Committee. Eighteen NativeSkin models were separated into two groups (6 non-tape-stripping and 12 tape-stripping groups). The number of strippings was 10, 30, and 50 (*n* = 3 per group). NativeSkin models were cultured in a 5% CO_2_ incubator at 37 °C for 24 h. On days 0, 2, and 4, 6 µg/cm^2^ of PM_2.5_ in phosphate-buffered saline (PBS) was treated on the surface of the NativeSkin models. On day 6, the skin tissues were washed with PBS three times and then measured by MLNO microscopy.

### 4.3. Multimodal Nonlinear Optical (MNLO) Imaging

We applied our previously developed MNLO imaging method [20,21,22]. Briefly, we used two separate laser systems: a picosecond-pulsed laser system (pico-EMERALD; HighQ Laser, APE, Berlin, Germany) for CARS imaging, and a femtosecond-pulsed laser system (Chameleon Vision-S; Coherent Inc., Santa Clara, CA, USA) for TPEF imaging. To obtain CARS signals corresponding to Raman peaks 1 and 2 of PM_2.5_ and SRM 2786, we set the center wavelengths of the pump beam to 827 nm and 817 nm, respectively, and the center wavelength of the Stokes beam to 1064 nm. To obtain TPEF and SHG signals, we set the wavelength of the fs-pulsed laser to 810 nm. All images were obtained using FluoView software (FV10-ASW; Olympus Corp., Tokyo, Japan). 

For *en face* 3D MNLO imaging, human skin biopsy samples were mounted with the stratum corneum (SC) region facing down on a coverglass-bottom chamber and then covered with a coverslip. z-depth images were obtained to measure depths of 0–150 μm. For side-view imaging, samples were cross-sectioned into 50 μm thick slices.

### 4.4. Enzyme-Linked Immunosorbent Assay for Pro-Inflammatory Cytokines

To measure pro-inflammatory cytokines, 6 µg/cm^2^ of PM_2.5_ in PBS was applied on the surface of the human skin explant models for 24 h, after which the tissue culture medium was harvested for enzyme-linked immunosorbent assay (ELISA). The secretion levels of the pro-inflammatory cytokines (IL-1α, IL-1β, TNFα, and IL-6) were measured using a Human Cytokine/Chemokine Magnetic Bead Panel (#HCYTOMAG-60K, Merck-Millipore, Burlington, MA, USA) according to the manufacturer’s instructions.

### 4.5. Immunofluorescence Imaging

For immunofluorescence imaging, cross-sectioned slices of human skin biopsy samples were fixed with 4% paraformaldehyde for 15 min. After washing three times with 1× PBS containing 0.1% Triton X-100 (Sigma Aldrich, St. Louis, MO, USA), the samples were permeated and blocked with a 1× PBS solution containing 5% normal horse serum and 0.5% Triton X-100 for an additional 90 min. IL-1α (MAB200, R&D Systems, Minneapolis, MN, USA) and IL-1β (ab9722, Abcam, Cambridge, MA, USA) antibodies were incubated with the samples overnight at 4 °C. After washing three times with 1× PBS containing 0.1% Triton X-100, secondary antibodies conjugated with Alexa Fluor 488 (A21206; Thermo Fisher Scientific, Waltham, MA, USA) and Alexa Fluor 594 (A11007; Thermo Fisher Scientific, Waltham, MA, USA) were incubated with the samples for 60 min at room temperature. Counter-staining was performed with 1 μg/mL DAPI for 3 min at room temperature. Images were obtained using confocal microscopy (FV3000, Olympus Corp., Tokyo, Japan).

## 5. Conclusions

We have developed a suitable methodology for human dermatological investigations using MNLO imaging. We point out the strength of this system is that it can cover investigations of not only PM but also other factors by assigning the given molecular specificities simultaneously with the anatomical information of the skin. The results may help to identify the effects of various factors on human skin, which will benefit the development of therapeutic interventions in the future.

## Data Availability

Not applicable.

## Supplementary Materials

The following are available online at https://www.mdpi.com/article/10.3390/ijms22105199/s1: Supplementary Figure S1: *En face* MNLO imaging of human skin biopsy samples treated with PAH, Supplementary Video S1: Visualization of PM_2.5_ in human skin by *en face* 3D MNLO imaging, Supplementary Video S2: Visualization and analysis of PM_2.5_ penetration into human skin by MNLO imaging.
