# Supplementary material for: Molecule-Resolved Visualization of Particulate Matter on Human Skin Using Multimodal Nonlinear Optical Imaging
Source: Int J Mol Sci. 2021 May 14;22(10):5199. doi: 10.3390/ijms22105199 (PMC8156198; doi:10.3390/ijms22105199)
Supplement: Supplementary file 1 [file ijms-22-05199-s001.zip › ijms-1208058-supplementary.pdf]

Supplementary Materials

Molecule-resolved visualization of particulate matter on human skin using multimodal nonlinear optical imaging

Eun-Soo Lee[1†], Suho Kim[2,3†], Sang-Won Lee[3,4], Jinsang Jung[5], Sung Hoon Lee[1], Hye-Won Na[1], Hyoung-June Kim[1], Yong Deog Hong[1], Won Seok Park[1], Tae Geol Lee[3], Dong-Gyu Jo[2], and Se-Hwa Kim[3,4*]

[1]AMOREPACIFIC Research and Development Center, Yongin 17074, Rep. of Korea

[2]School of Pharmacy, Sungkyunkwan University, Suwon 16419, Rep. of Korea

[3]Safety Measurement Institute, Korea Research Institute of Standards and Science, Daejeon 34113, Rep. of Korea

[4]Department of Medical Physics, University of Science and Technology, Daejeon 34113, Rep. of Korea

[5]Gas Metrology Group, Division of Chemical and Biological Metrology, Korea Research Institute of Standards and Science, Daejeon 34113, Rep. of Korea

Contents

Supplementary Figure S1. *En face* MNLO imaging of human skin biopsy samples treated with PAH.

Supplementary Video S1. Visualization of $PM_{2.5}$ in human skin by *en face* 3D MNLO imaging.

Supplementary Video S2. Visualization and analysis of $PM_{2.5}$ penetration into human skin by MNLO imaging.

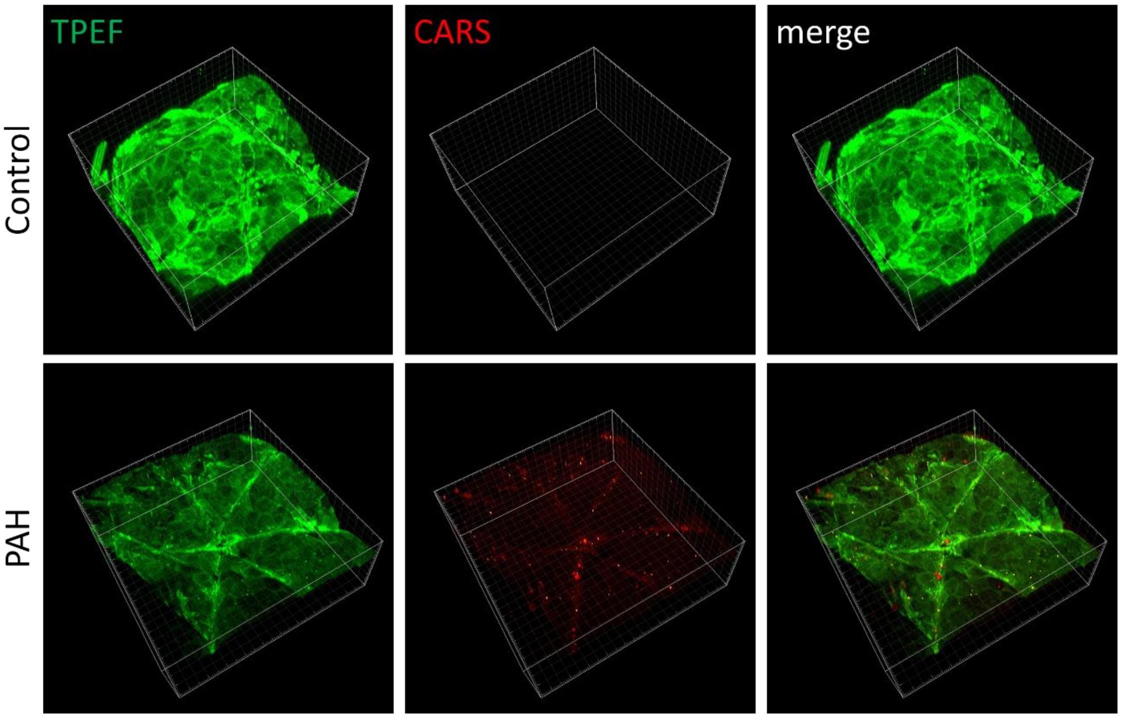

Supplementary Figure S1. *En face* MNLO imaging of human skin biopsy samples treated with PAH. 0.6 µg/ml of PAH- or vehicle-treated samples for 24 h. TPEF signals (green) were measured at Ex.810 nm/Em. 495–540 nm and C–C CARS signals (red) were obtained at 2693 cm^{-1}. Measurement volume was 420 (x) x 420 (y) x 200 (z) μm^3.

Supplementary Video S1. Visualization of $PM_{2.5}$ in human skin by *en face* 3D MNLO imaging. *En face* MNLO images of the label-free stratum corneum (TPEF, green) and $PM_{2.5}$ (CARS, red in upper panel and yellow in lower panel) in human skin biopsy samples after $PM_{2.5}$ exposure. Consecutive *en face* MNLO image slices were reconstructed in 3D.

Supplementary Video S2. Visualization and analysis of $PM_{2.5}$ penetration into human skin by MNLO imaging. MNLO images of label-free elastin and keratin (TPEF, green), $PM_{2.5}$ (CARS, red), and collagen (SHG, magenta) in cross-sectioned $PM_{2.5}$-exposed human skin biopsy samples after 50 strippings. Consecutive *en face* MNLO image slices were reconstructed in 3D.
